# Beyond Folding: Expanding the Functional Landscape of Hsp90 Chaperone Machinery in Health and Disease

**DOI:** 10.3390/ijms262110279

**Published:** 2025-10-22

**Authors:** Manish Kumar Singh, Jyotsna S. Ranbhise, Minghao Fu, Songhyun Ju, Sunhee Han, Hyeong Rok Yun, Wonchae Choe, Sung Soo Kim, Insug Kang

**Affiliations:** 1 Department of Biochemistry and Molecular Biology, School of Medicine, Kyung Hee University, Seoul 02447, Republic of Korea; manishbiochem@gmail.com (M.K.S.); jogm25@khu.ac.kr (J.S.R.); andrew1179609214@gmail.com (M.F.); thdgus8543@khu.ac.kr (S.J.); sunheehan@khu.ac.kr (S.H.); foryou018@naver.com (H.R.Y.); wchoe@khu.ac.kr (W.C.); 2 Biomedical Science Institute, Kyung Hee University, Seoul 02447, Republic of Korea; 3 Department of Biomedical Science, Graduate School, Kyung Hee University, Seoul 02447, Republic of Korea

**Keywords:** Hsp90, ATPase, client proteins, co-chaperone, cancer, cardiac disease, diabetes

## Abstract

Molecular chaperones are crucial for maintaining protein homeostasis by assisting in the proper folding, stabilization, and function of proteins. Among them, Heat shock protein 90 (Hsp90), represents a highly conserved protein family of molecular chaperones that plays an essential role in diverse biological processes and is fundamental to cellular health and survival. As a highly abundant molecular chaperone, Hsp90 comprises 1–2% of cellular proteins, increasing to 4–6% under stress conditions. It interacts with client proteins, assisting them in proper folding and stability. Unlike classical chaperonins, Hsp90 operates through a highly regulated, ATP-dependent cycle that involves multiple co-chaperones. This process allows Hsp90 to selectively engage with numerous client proteins, including signaling proteins, kinases, hormone receptors, and transcription factors. Recent discoveries have revealed its involvement in processes beyond protein folding, demonstrating its role in diverse cellular functions such as epigenetic regulation, immune signaling, and oncogenic transformation. This current review highlighted the specific characteristics of cytoplasmic and endoplasmic reticulum (ER) as well as mitochondrial paralogs and functions, focusing on its contribution to buffering genetic variation, facilitating oncogene addiction, and modulating disease phenotypes in conditions such as cancer, neurodegeneration, cardiovascular diseases (CVD), and diabetes. We also discuss the therapeutic potential of targeting Hsp90 and its co-chaperones, outlining the challenges and prospects in drug development. These insights not only reshape our understanding of chaperone biology but also present opportunities for precision medicine in various human diseases.

## 1. Introduction

The heat shock protein 90 (Hsp90), is a highly abundant and conserved molecular chaperone which plays an essential role in maintaining protein homeostasis across all organisms. In eukaryotic cells, Hsp90 is essential for cell viability under both normal and stress conditions. Among the heat shock proteins (HSPs), Hsp90 is the most prominent chaperone, accounting for approximately 1–2% of total cellular protein under normal physiological conditions, with levels increasing to 4–6% during stress [1]. Hsp90 comprises four main isoforms: the inducible cytosolic Hsp90α (Hsp90AA1), the constitutive cytosolic Hsp90β (Hsp90AB1), the ER resident Grp94/Gp96, and the mitochondrial paralog tumor necrosis factor receptor-associated protein-1 (TRAP1) [2]. Additionally, there is an alternative splice variant, Hsp90N, which is associated with cellular transformation. TRAP1 shares similarities with the bacterial homolog HtpG and is found in the mitochondrial matrix, while ER-resident Hsp90 is localized in the ER lumen (Table 1). This indicates a close evolutionary relationship between TRAP1 and eubacterial HtpG [3]. In plants, Hsp90 has been identified in the chloroplast (cHsp90). It plays a role in the import of proteins into the organelle as well as in the maturation of proteins related to photosynthesis [4].

### 1.1. Hsp90 Structure and Co-Chaperone and Client Interactions

**Figure 1:**
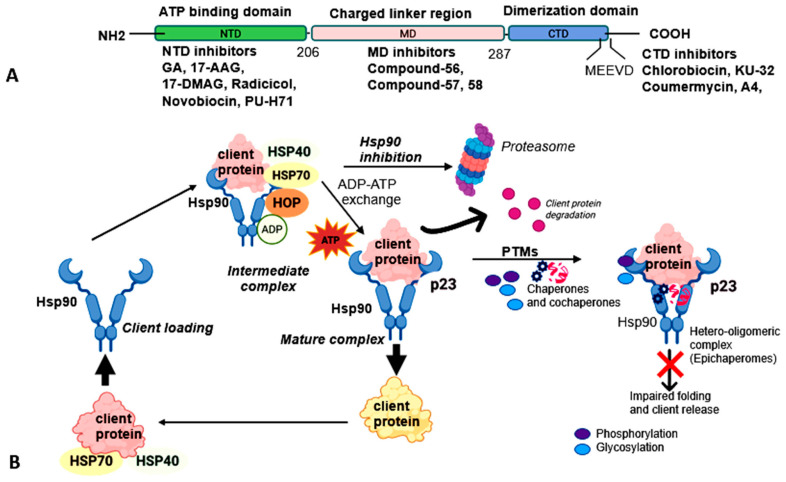
Schematic representation of structural organization of Hsp90 and its interactions with co-chaperones and client proteins. (**A**) Organization of human Hsp90 gene and its functional domains: N-terminal domain (NTD), middle domain (MD), and C-terminal domain (CTD) (adapted from the NCBI Gene database). (**B**) Illustration of the Hsp90 chaperone cycle, showing how ATP binding and hydrolysis drive conformational changes between open and closed states, facilitating the recruitment, stabilization, and maturation of co-chaperones and client proteins. The post-translational modifications (PTMs) promote the formation of hetero-oligomeric complex and inhibit the release of client proteins.

Hsp90 is a cytosolic molecular chaperone that primarily exists as an elongated homodimer. In mammalian cells, two cytosolic isoforms of Hsp90 have been identified: Hsp90α and Hsp90β, encoded by distinct genes and sharing approximately 84% amino acid sequence identity [2]. In contrast, the bacterial homolog HtpG is largely dispensable under non-stress conditions [11]. In *S. cerevisiae*, two Hsp90 isoforms, Hsc82 and Hsp82, share 97% amino acid sequence homology. Hsc82 is constitutively expressed, whereas Hsp82 is induced in response to stress [12]. Higher eukaryotes contain both homodimers (α-α, β-β) and heterodimers (α-β) [13]. Each Hsp90 monomer consists of three structural domains: an N-terminal nucleotide-binding domain (NTD) responsible for ATP binding and hydrolysis, a middle domain (MD) that interacts with client proteins, and a C-terminal dimerization domain (CTD) featuring a conserved MEEVD recognition motif [14,15]. The N-terminal domain contains the canonical ATP-binding pocket, which also serves as a target for several natural product inhibitors, such as radicicol and geldanamycin. These compounds interfere with Hsp90’s function, destabilizing protein kinases and ultimately promoting their degradation [16]. The MD, comprising approximately 50 amino acid residues, is crucial for coordinating interactions between the NTD and CTD, linking ATP binding with conformational changes in Hsp90 [17]. Notably, the MD has a catalytic site that binds the γ-phosphate of ATP, classifying Hsp90 as a “split ATPase”. Structural similarities with DNA topoisomerase II (gyrase B) further underscore the MD’s significance as a primary docking site for client proteins such as AKT, Cdc37, p53, and eNOS [18,19,20]. Recent studies have revealed that human Hsp90 (hHsp90) adopts a more flexible structural confirmation, predominantly in an open/apo state. This structural plasticity allows Hsp90 to accommodate a broader range of clients and co-chaperones, providing additional regulatory mechanisms (Figure 1A,B) [21].

Moreover, the modulation of lid dynamics in hHsp90 represents an evolutionary adaptation that alters its functional properties and cellular requirements [22]. In vivo, Hsp90 function is regulated by a repertoire of co-chaperones that either directly modulate client proteins or fine-tune the chaperone cycle, resulting in a highly dynamic network of transient chaperone and co-chaperone assemblies [23]. Proteome-wide analyses have revealed that approximately 10% of human proteins are clients of Hsp90, and their proper folding and maturation critically rely on it. These client proteins encompass diverse functional categories, including transcription factors, ubiquitin-related proteins, and a significant number of kinases. Notably, nearly 60% of Hsp90-dependent kinases are part of the serine/threonine kinase family, such as Cdc37, eIF-2α, Src kinases, and Raf kinases. Hsp90 plays a crucial role in regulating essential cellular processes, such as signal transduction, apoptosis, and cell survival, through its interactions with these proteins [24,25]. Hsp90 co-chaperones exhibit substantial structural diversity, and their interactions are dynamically modulated by cofactors such as activator of Hsp90 ATPase homolog 1 (Aha1), Hop, Cdc37, and p23. Several co-chaperones regulate the ATPase activity of Hsp90 in a manner that is specific to both the client and the cellular context. For instance, Aha-1 and high copy Hsp90 suppressor 1 (Hch1), directly stimulate the basal ATPase activity of Hsp90 [26]. The interaction of the GR ligand-binding domain with Hsp90 has been shown to enhance Hsp90 ATPase activity by nearly 200-fold [27]. In contrast, other clients, including the mineralocorticoid receptor ligand-binding domain (MR-LBD), Tau, and the p53 DNA-binding domain (DBD), do not significantly alter Hsp90 ATPase activity [28]. Recognition of client proteins by Hsp90 is driven by conformational rearrangements that expose specific binding interfaces. While the initial modes of interaction vary among different clients, they ultimately converge on the formation of productive closed Hsp90 complexes. ATP-dependent allosteric transitions mediate communication between Hsp90 domains, a process further fine-tuned by co-chaperones such as p23, thereby adapting the chaperone cycle in a client-specific manner [28].

**Figure 2:**
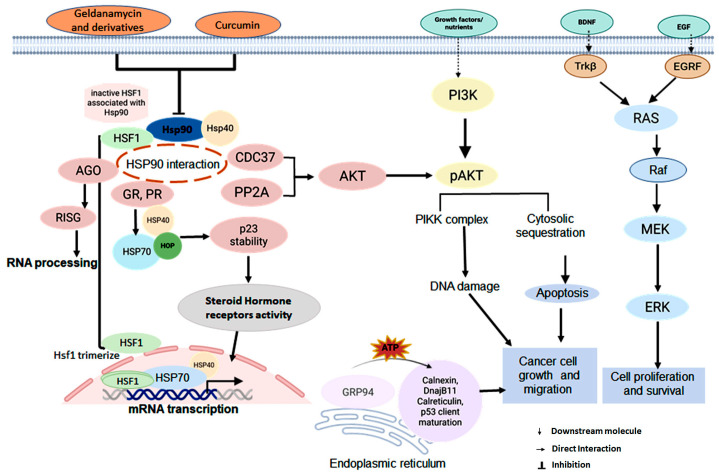
The schematic illustration of Hsp90 interactions with signaling pathways and client protein kinases. Schematic representation of Hsp90 involvement in diverse cellular pathways, including cancer, steroid hormone signaling, and cell death. In oncogenic signaling, Hsp90 associates with key client proteins, such as AKT, Ras/Raf, p23, ATM, ATR, and HSF1—supporting downstream cascades that promote tumor cell growth, survival, and adaptation.

Another co-chaperone such as Hop/Sti1 possesses TPR motifs that allow simultaneously interaction with both Hsp70 and Hsp90, which stabilizes an open confirmation of Hsp90 and inhibits its ATPase activity [29]. Another TPR-containing co-chaperone, PP5/Ppt1, regulates the Hsp90 cycle by dephosphorylation of Hsp90 and the co-chaperone Cdc37, thus affecting the client maturation [30]. Other TPR-containing co-chaperones, including FK506-binding proteins such as FKBP52 (FKBP4), cyclophilin 40 (CPY40), as well as Cpr6 and Cpr7 in yeast, also modulate Hsp90 function through their peptidyl-prolyl cis-trans isomerase (PPIase) domains. Cdc37, which is specific to kinases, interacts with both client kinases and the NTD of Hsp90, partially inhibiting Hsp90’s ATPase activity. In contrast, p23/Sba1 binds directly to the NTD, stabilizing the closed conformation of Hsp90, which reduces ATPase activity and regulates the progression of the chaperone cycle and maturation of client proteins [31]. Beyond protein folding, the Hsp90-Hsp70 chaperone machinery is critical in RNA metabolism, particularly in loading small RNA duplexes into Argonaute (AGO) proteins during the assembly of the RNA-induced silencing complex (RISC). Pharmacological inhibition of Hsp90’s ATPase activity with geldanamycin and its derivatives significantly reduces RISC activity, underscoring the essential role of Hsp90 in small RNA processing pathways (Figure 2) [32].

Most client proteins are recruited via large multiprotein complexes formed by the Hsp90/Hsp70 chaperone machinery [33]. Under stress conditions, cells activate protective mechanisms by either inducing pro-survival kinases or inhibiting pro-apoptotic signaling pathways [34]. AKT is a well-characterized Hsp90-dependent kinase, whose active conformation is stabilized via intracellular complex formation with Cdc37. Hsp90 inhibition suppresses AKT phosphorylation, leading to increased apoptosis. In interstitial epithelial cells (IECs) exposed to hypoxic conditions, a reduction in Hsp90 decreases phosphorylated AKT (pAKT), triggering apoptosis, whereas an increase in Hsp90 enhances pAKT levels and reduces apoptosis. This highlights the role of AKT in Hsp90-dependent cell survival [35]. Specifically, Hsp90β protects hypoxic IECs from apoptosis by enhancing the activity of the pro-apoptotic protein Bad, which in turn reduces the release of cytochrome c, caspase-3 activation, and PARP cleavage [36]. Additionally, Hsp90 promotes AKT activity by inhibiting protein phosphatase 2A (PP2A) [18]. Emerging biophysical approaches, such as single-molecule fluorescence and force spectroscopy, are improving our understanding of these dynamic interactions (Figure 2) [37].

The stability of Hsp70-based complexes is further enhanced by Hsp70-interacting proteins (HIP), which facilitate ATP hydrolysis. For instance, Hsp40 initiates the chaperoning of progesterone receptor (PR) by regulating the ATPase activity of Hsp70 [38]. The PR then forms a complex with Hsp40 and Hsp70, which subsequently recruits Hop and Hsp90, resulting in formation of an intermediate complex. This complex ultimately matures into a PR: Hsp90: p23 complex. The co-chaperone p23 selectively binds to Hsp90 in its ATP-bound state, interacting with both the N-terminal and middle domains of dimeric Hsp90 [39]. This interaction stabilizes the closed conformation of Hsp90 and maintains the receptor’s ligand-binding domain in a configuration that allows for high-affinity hormone binding [40]. Characterizing the binding interfaces of Hsp40 on PR and exploring whether similar sites exist on other Hsp90 client proteins represents an important avenue for future research. A comprehensive list of Hsp90 interactors is available on the following link https://www.picard.ch/downloads/Hsp90interactors.pdf (accessed on 27 August 2025).

The glucocorticoid receptor (GR), a prototypical member of the steroid hormone receptor (SHR) family, requires interaction with Hsp90 to attain its hormone-binding conformation [41]. The interaction of GR and Hsp90 is ATP-dependent process that involves a prior activity of Hsp70 and the co-chaperone Hop, leading to the formation of an inactive “loading complex” comprising GR, Hsp90, Hsp70, and Hop. upon ATP hydrolysis by Hsp90, Hsp70, and Hop, the latter two components are released, permitting the incorporation of p23 and the assembly of the active GR: Hsp90: p23 “maturation complex”, which restores GR ligand-binding activity with enhanced affinity [42]. Notably, the C-terminal tail helix of p23 can directly interact with the GR ligand-binding domain (GR-LBD) in vitro, thereby promoting receptor maturation even in the absence of Hsp90 [43]. The highly conserved hydrophobic groove of GR interacts with the p23 tail helix, stabilizing the closed confirmation of Hsp90 and underscoring the critical role of p23-dependent chaperoning across all SHRs [44]. The conformational transition of the Hsp90 complex from an open-to-closed state is coordinated by ATPase activities of both Hsp70 and Hsp90 [45,46]. Recent Cryo-EM studies have revealed that GR binds within the lumen of Hsp90, with the complex further stabilized by p23 [44].

### 1.2. ER-Resident Hsp90 (Grp94)

Glucose-regulated protein 94 (Grp94, also known as gp96 or Hsp90B1) is an ER-resident paralog of Hsp90 that shares approximately 50% sequence homology with its cytoplasmic counterpart [47]. Like Hsp90s, Grp94 functions as a homodimer, composed of two identical promoters comprising an NTD, MD, and a CTD. A unique feature of Grp94 is the presence of a pre-N domain within the NTD, which regulates ATP hydrolysis and the maturation of client protein [48]. Grp94 exhibits a shorter, lysine-rich charged linker that contains multiple calcium-binding sites [49]. It also possesses a carboxy-terminal KDEL motif, which facilitates its retention in the ER, and it lacks the canonical carboxy-terminal MEEVD sequence. Due to its hydrophobic properties, Grp94 is associated with the membrane of the ER and Golgi complex [50]. Grp94 interacts with a variety of ER-resident proteins, including protein kinases, calmodulin, and molecular chaperones such as Grp78 (BiP), calnexin, calreticulin, and Grp170. The interaction between Grp94 and BIP is client-specific and does not exhibit a general chaperone mechanism. In vivo, the maturation of Toll-like receptors (TLRs) requires the client-specific co-chaperone canopy fibroblast growth factor signaling regulator 3 (CNPY3). CNPY3 forms a trimolecular complex with gp96 and TLR9 precursor 1, thereby synergistically promoting the tertiary folding of the TLR9 precursor 2. Once the TLR9 precursor 2 achieves its properly folded conformation, it dissociates from the gp96–CNPY3 complex and is subsequently translocated to the endolysosome for further processing [51]. These findings suggest that the interaction between Grp94 and the ER-resident chaperone BiP may become particularly critical in the absence of client-specific co-chaperones [52].

Grp94 is involved in protein folding, processing, and secretion, and it plays a significant role in the signaling pathways of steroid hormones, integrins, and TLRs, which are crucial for immunity and metastasis. ATP binding induces conformational changes in the lid region of Grp94, while BIP binding stabilizes a partially closed Grp94 intermediate. Conversely, impaired ATP binding disrupts the processing of client proteins. Under conditions of ER stress, Grp94 can adopt an ATP-independent “holdase” mode, stabilizing unfolded proteins until normal folding conditions are restored. This ATP-independent activity has been observed in the co-chaperone-independent processing of p53 and the stabilization of the client kinase v-Src under heat stress [53,54].

**Figure 3:**
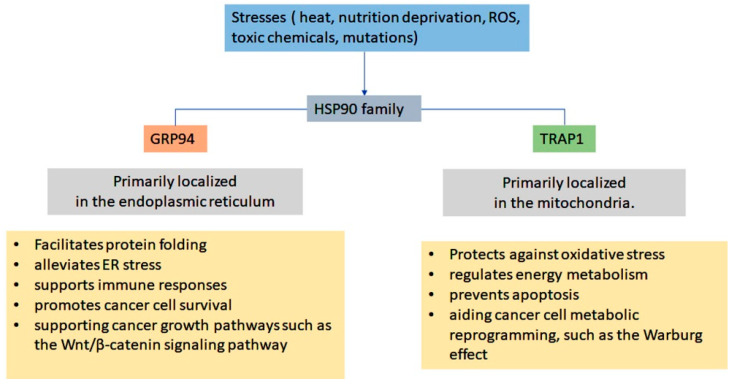
The schematic representation of organelle-specific Hsp90 homologs in cancer and oxidative stress. Schematic representation of ER and mitochondrial specific Hsp90 functions in diverse cancers and reactive oxygen species (ROS) regulation, highlighting the roles of its endoplasmic reticulum (Grp94) and mitochondrial (TRAP1) homologs under stress conditions. The figure also depicts ER- and mitochondria-mediated processes that support cancer cell growth, survival, and metastasis.

An emerging regulatory paradigm in chaperone biology is the concept of the “chaperone code,” wherein post-translational modifications (PTMs) dynamically modulate chaperone activity and client interactions [55]. Proteomic analyses have identified nearly 300 PTM sites on Grp94, most of which localize within the pre-N domain, suggesting a complex and largely uncharacterized layer of regulation. Functionally, Grp94 is indispensable for the stability and maturation of multiple cancer-associated proteins, including TLRs, insulin-like growth factors (IGFs), immunoglobulins, and p53 [56]. For instance, the E3 ubiquitin ligase FBXL2, a member of the conserved F-box protein family, targets both EGFR and tyrosine kinase inhibitor (TKI)-resistant EGFR mutants for proteasome-mediated degradation in EGFR-driven non-small cell lung cancer (NSCLC). FBXL2 itself serves as a client of Grp94, competing with EGFR for Grp94 binding. This competition can alter protein conformations, thereby disrupting the stable interaction between FBXL2 and EGFR. Consequently, the combined strategy of Grp94 inhibition and FBXL2 activation has been shown to exert potent growth-suppressive effects in EGFR-TKI-resistant NSCLC models [57]. Knockdown studies further implicate Grp94 in cell adhesion and migration, providing mechanistic insights into its role in regulating cancer cell motility (Figure 3) [58]. Despite its importance, the cellular functions of Grp94 remain less understood compared to cytosolic or mitochondrial Hsp90. Collectively, these findings highlight Grp94 as a central regulator of proteostasis and signaling in the ER, with particular relevance in cancer biology. Its unique structural features, ATP-dependent and -independent activities, and PTM-driven regulation underscore its therapeutic potential, warranting further exploration of client interactions and regulatory mechanisms for drug discovery.

### 1.3. Mitochondrial Hsp90 (TRAP1)

TRAP1, also known as Hsp75, is a mitochondrial molecular chaperone that plays a pivotal role in maintaining protein homeostasis and preserving mitochondrial integrity under cellular stress conditions. TRAP1 shares approximately 34% amino acid sequence identity with Hsp90β and contains an N-terminal mitochondrial targeting sequence that is cleaved upon import into the mitochondrial matrix. Unlike cytosolic Hsp90 isoforms, TRAP1 lacks the highly charged, flexible linker region typically located between the N- and middle (M) domains [7] as well as the C-terminal MEEVD motif required for the recruitment of co-chaperones. Consequently, TRAP1 can function independently of classical Hsp90 co-chaperones such as p23 and Hop [59]. The binding of client proteins to TRAP1 requires a confirmation transition into the open form. Clients are directly loaded via direct protein–protein interactions, which subsequently promote ATP binding and hydrolysis, driving conformational rearrangements that facilitate client remodeling [60]. Sequential ATP hydrolysis events may generate structural strain on client proteins positioned between TRAP1 protomers, thereby assisting their refolding and stabilizing their native conformations or enabling the formation of multiprotein complexes. Conversely, inhibition of TRAP1 with ATP-mimetic compounds such as Gamitrinibs (geldanamycin-based conjugates) or SMTIN-P01 leads to proteolytic degradation of unfolded client proteins [61]. Gamitrinibs rapidly accumulate within mitochondria, where they compromise mitochondrial integrity by influencing the mitochondrial permeability transition pore (mPTP), promoting cytochrome c release, and activating caspases in prostate cancer cells [62]. These findings highlight the therapeutic significance of mitochondria-targeted drugs as promising anticancer strategies.

Under certain conditions, client proteins bound to TRAP1 do not undergo classical folding but instead achieve structural stabilization through TRAP1’s “holdase” activity. For instance, the interaction between TRAP1 and SIRT3 stimulates ATPase activity, indicative of this holdase function, which enhances the enzymatic activity of client proteins. However, due to the absence of co-chaperones, e.g., p90, TRAP1 is unable to mediate the maturation of the progesterone receptor (PR). In contrast, cytosolic Hsp90 facilitates PR maturation through its cooperation with co-chaperones such as Hop and p23, a process primarily carried out by the Hsp90α and Hsp90β isoforms [63]. A distinct mitochondrial pool of Hsp90 has been identified in various cancer cells as well as in certain normal mouse tissues. This mitochondrial Hsp90 population appears to be regulated differently from its cytosolic counterpart and does not physically interact with TRAP1 [8]. Beyond its chaperoning function, TRAP1 regulates multiple mitochondrial processes, including bioenergetics, redox homeostasis, oxidative stress responses, mitochondrial dynamics, mitophagy, and resistance to apoptosis [64].

Functionally, TRAP1 exhibits a dual role: it acts as a cytoprotective factor under physiological stress while also promoting tumor progression. The metabolic reprogramming of cancer cells from oxidative phosphorylation (OXPHOS) to glycolysis, a hallmark of tumor metastasis is partially regulated by TRAP1 through its interaction with succinate dehydrogenase alpha (SHDα) under hypoxic and nutrient deficient conditions [65]. During nutrient deprivation, TRAP1 enhances glutamine metabolism to meet elevated energy demands, thereby supporting cancer cell survival and proliferation [66]. Conversely, TRAP1 overexpression and SDH inhibition can protect tumor cells from oxidative stress by preventing the opening of the mitochondrial permeability transition pore (mPTP), inhibiting cytochrome c (Cyt c) release, and suppressing apoptosis [67]. Cyclophilin D (CypD), another regulator of mPTP opening, maintains mitochondrial Ca^2+^ homeostasis. Excessive Ca^2+^ accumulation disrupts F1F0-ATP synthase activity, promotes ROS generation, and triggering mPTP opening, loss of mitochondrial membrane potential, and apoptosis. TRAP1 induction inhibits CypD-dependent mPTP opening, thereby preventing the mitochondrial-mediated apoptosis [68]. Moreover, TRAP1 stabilizes HIF1α via SDH inhibition, promoting the downstream genes that enhance angiogenesis and nutrient supply in proliferating tumor cells [69]. TRAP1 prevents the ubiquitin-mediated degradation of CDK1 and MAD2, supporting mitotic progression in cancer cells. This regulatory mechanism has been demonstrated in breast, colorectal, and lung carcinoma cell lines and tumor samples, highlighting TRAP1 as a promising therapeutic target in these malignancies [70].

Mitochondria-targeted Hsp90 inhibitors have emerged as promising agents for selectively modulating TRAP1 activity while minimizing off-target effects associated with pan-Hsp90 inhibition. The compound SMTIN-P01 demonstrated enhanced cytotoxicity against various cancer cell lines (22Rv1, A172, H460, and MDA-MB-231) compared with PU-H17, underscoring the therapeutic potential of mitochondrial targeting. Incorporation of a triphenylphosphonium (TPP) moiety facilitates the mitochondrial accumulation of these inhibitors and enables engagement with an allosteric site on TRAP1, thereby increasing its ATPase activity [61]. Similarly, the C-terminal inhibitor 6BrCaQ-C10-TPP, generated by linking 6BrCaQ to TPP through variable linkers exhibited potent nanomolar GI_50_ values against multiple cancer cell lines, including MDA-MB-231, HT-29, HCT-116, K562, and PC-3. This compound significantly affected mitochondrial membrane potential and preserved TRAP1 client proteins such as SDH without inducing a heat-shock response [71]. DN401, another TRAP1-selective inhibitor, also showed preferential binding and potent antiproliferative effects in cancer cells. Alternatively, the addition of cationic moieties such as TPP, rhodamine, cyanine, or cationic peptides into Hsp90 inhibitors enhances their mitochondrial accumulation by 100–1000-fold, as confirmed through fluorescence co-localization with MitoTracker dyes and mitochondrial isolation assays [72].

Natural compounds have also been identified as inhibitors of TRAP1. For instance, Honokiol lipophilic bisdichloroacetate ester (HDCA) exhibits potent antineoplastic activity by inducing oxidative stress and apoptosis through selective inhibition of TRAP1 ATPase activity at low micromolar concentrations, without affecting cytosolic Hsp90 isoforms [73]. Similarly, green tea extract (GTE) and its major polyphenolic component, epigallocatechin gallate (EGCG), broadly inhibit chaperone activity. Both GTE and EGCG suppress the expression of TRAP1, Hsp90, and Hsp27, thereby impairing tumor growth and promoting apoptosis [74]. Collectively, these findings indicate that both HDCA and GTE exert anticancer effects through inhibition of mitochondrial and cytosolic heat shock proteins, positioning TRAP1 as a promising therapeutic target for cancer treatment [65].

### 1.4. Post Translational Modifications (PTMs)

PTMs such as phosphorylation, acetylation, glycosylation, methylation, ubiquitination, and SUMOylation of Hsp90 have been identified as vital regulators of its interactions with co-chaperones and client proteins [75]. These modifications collectively regulate Hsp90 conformational dynamics, the maturation of client proteins, and interactions with co-chaperones [76]. Phosphorylation of Hsp90α at Thr90 by protein kinase A alters its interactions with cofactors and client proteins. This modification enhances binding to ATP and cochaperones such as Aha1, p23, PP5, and CHIP, while reducing association with Hsp70, Hop, and Cdc37. It also impairs the binding of client kinases including Src, Akt, and PKCγ. Notably, Thr90 phosphorylation is elevated in proliferating cells, suggesting a regulatory role in tumor metastasis [77]. Another prominent phosphorylation sites at serine residues Ser226 and Ser255, with additional sites found on tyrosine. This modification affects Hsp90’s conformational transitions, thereby influencing client protein folding and Hsp90 conformational transitions, modulating client protein folding and co-chaperone binding. The phosphorylation close to the TPR recognition site located in the CTD at Thr725 and Ser726 are adjacent phosphorylation, which influence the co-chaperone interaction [78]. Particularly, hyperphosphorylation disrupts these processes, whereas basal phosphorylation promotes Hsp90 dimerization and facilitates the assembly of higher-order oligomers [79].

Additionally, phosphorylation of Hsp90β at Ser226 and Ser255 stabilizes a closed conformation by orienting the linker in an upward direction, thereby exposing the middle domain of Hsp90 and promoting stable interactions with co-chaperones such as [80]. Methylation of Hsp90 at lysine 616 (K616) by Smyd2 is required for proper sarcomere function; however, this observation still requires validation in cardiac and skeletal muscle cells [81]. Additionally, S-nitrosylation of Hsp90 at cysteine 589 (C589) by nitric oxide (NO) stabilizes the TGF-β receptor and mitigates fibrosis by blocking the TGF-β/SMAD3 signaling pathway in cardiomyocytes [82]. Another report showed that SUMOylation of Hsp90 at lysine 191 (K191) has been shown to facilitate recruitment of the co-chaperone Aha1, which enhances Hsp90 ATPase activity [83], whereas Hsp90α-K283, and Hsp90β-K275 modulates drug binding to Hsp90 by influencing both adjacent and distal serine and threonine phosphorylation sites [84]. These conformational rearrangements disrupt Hsp90’s canonical ATPase-driven folding cycle but promote its incorporation into higher-order, multimeric epichaperome assemblies. Under stress conditions, N-glycosylation of Grp94 at Asn62, located within intrinsically disordered regions (IDRs), stabilizes a pathological conformation that facilitates its integration into epichaperomes [85]. In breast cancer, the Grp94 N62Q mutant, which fails to undergo glycosylation, exhibits reduced epichaperome formation, diminished plasma membrane localization, and attenuated EGFR signaling compared to N217A mutant, which retain EGFR signaling. This demonstrates that Grp94 glycosylation is critical for the pathological induction and stabilization of epichaperomes via extensive protein–protein interactions (PPIs). For instance, over 2481 proteins were sequestered in breast cancer cells associated with aggressive phenotypes. Notably, cells entering mitosis in the presence of epichaperome disruption fail to complete cell division and ultimately undergo cell death, underscoring their dependency on epichaperome-mediated mitotic rewiring [84]. In summary, these findings suggest that targeting epichaperomes to restore context-specific PPI network function may represent a promising therapeutic strategy for diseases such as cancer and Alzheimer’s disease (AD) [86].

### 1.5. Role of Hsp90 in Cellular Processs and Protein Homeostasis

Hsp90 has been reported in various cellular processes including protein folding, signal transduction, maturity and stability of a wide range of client proteins [87]. These client proteins encompass steroid hormone receptors, transcriptional factors, signaling kinases, and growth factors [88]. In humans, Hsp90 exerts a significant regulatory influence on kinase activity by facilitating the assembly of client proteins into multimeric complexes, such as those involving kinases and kinetochore components, and by promoting ligand binding, as observed in steroid hormone receptors [89]. The extent of Hsp90’s direct participation in de novo folding or refolding of denatured polypeptides remains a topic of debate. In *S. cerevisiae*, loss of Hsp90 function does not result in widespread folding defects [90,91]. Similarly, in vitro studies indicate that Hsp90 alone is insufficient to refold denatured proteins. Instead, it stabilizes non-native substrates in a “folding-competent” state, thereby allowing other factors to facilitate subsequent folding [50].

**Figure 4:**
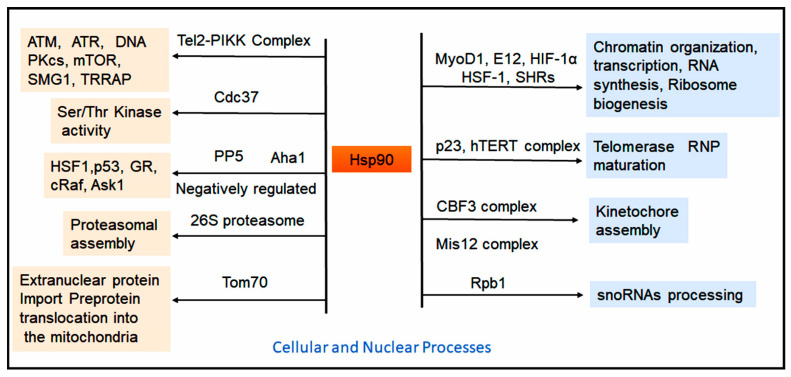
Schematic illustration of Hsp90-mediated cellular and nuclear processes. Hsp90 interacts with co-chaperones such as Cdc37, PP5, Aha1, and Tom70 to regulate diverse client proteins and complexes. In the cytoplasm, Hsp90 supports Ser/Thr kinases (ATM, ATR, DNA-PKcs, mTOR, SMG1, TRRAP), transcription factors (HSF1, p53, GR), and proteasomal assembly, as well as mitochondrial protein translocation. In the nucleus, Hsp90 facilitates the assembly and function of complexes involved in chromatin organization, transcription, RNA synthesis, ribosome biogenesis (MyoD1, E12, HIF-1α, HSF-1, SHRs), telomerase maturation (p23, hTERT), kinetochore assembly (CBF3, Mis12), and snoRNA processing (Rpb1).

More than half of all human kinases require Hsp90 and its co-chaperone Cdc37 for proper folding and activation. Hsp90 also modulates kinase activity by influencing PTMs. For instance, the Hsp90-associated serine/threonine-protein phosphatase 5 (PP5) dephosphorylates and inactivates c-Raf [92]. PP5, a member of the PPP phosphatase family, contains an N-terminal-TPR domain preceding its catalytic domain and acts as a negative regulator of several Hsp90 clients, including apoptosis signal–regulating kinase 1 (Ask1) under oxidative stress [93]. Other Hsp90 client kinases include ATM, which is activated in response to DNA double-strand breaks, and Chk1, which responds to UV-induced DNA damage [94,95]. The co-chaperone Aha1 binds to the MD of Hsp90, stimulating ATP hydrolysis and inorganic phosphate release. This activity promotes client folding, dissociation of co-chaperones and immunophilins, and resetting of Hsp90 to its open conformation [96]. PP5 also regulates several transcriptional regulators, such as Hsf1, p53, and the GR in an Hsp90-dependent manner (Figure 4) [97,98]. Additionally, phosphorylation of Cdc37 at Ser13 facilitates client recruitment to the Hsp90:Cdc37:kinase complex, while PP5-mediated dephosphorylation of Cdc37 within the complex promotes client release and kinase modification [99]. Beyond kinases, approximately 30% of E3 ubiquitin ligases and 7% of the transcription factors, including p53, interact with Hsp90. An updated list of Hsp90 client proteins is available at https://www.picard.ch/downloads/Hsp90interactors.pdf (accessed on 27 August 2025). The TPR co-chaperone CHIP, which contains a U-box domain homologous to E4 ubiquitination factors, recognizes both Hsp90 and Hsc70/Hsp70, targeting chaperone-bound misfolded proteins for ubiquitination and degradation.

Although predominantly cytoplasmic, approximately 5–10% of Hsp90 resides within the nucleus under basal conditions, with nuclear localization markedly increased during cellular stress. Nuclear Hsp90 associates with nucleoli [100] and peri-chromatin ribonucleoprotein fibrils [101], facilitating the nuclear import of proteins such as FKBP52, steroid hormone receptors (SHRs), and kinases [102,103]. Notably, these import processes are independent of calcium, calmodulin, and ATP [104]. Hsp90 also interacts directly with DNA, RNA, and histones, thereby influencing chromatin organization, transcriptional regulation, and RNA synthesis. It promotes the subnuclear translocation of steroid receptors to chromatin [105] and forms complexes with various transcription factors, including zinc finger proteins, helix–loop–helix proteins, MyoD1, E12, hypoxia-inducible factor-1α (HIF-1α), and heat shock factor-1 (Hsf1) [50]. Reduction in Hsp90 levels activates Hsf1 in vitro [106,107], while pharmacological inhibition by geldanamycin (GA) derivatives induces Hsf1 activation in vivo, suggesting that Hsp90 represses Hsf1 activity under non-stress conditions. Under normal circumstances, Hsp90 forms a complex with Hsf1, which dissociates during stress, allowing Hsf1 to trigger the heat shock response [107]. Additionally, Hsp90 supports ribosome biogenesis by facilitating the nuclear export of the assembled 60S ribosomal subunit into the cytoplasm [108].

The role of Hsp90 in telomerase ribonucleoprotein (RNP) assembly and function is less clearly defined. The Hsp90–p23 complex has been reported to be indispensable for telomerase RNP maturation and activity [109]. Inhibition of Hsp90 by GA and its derivatives suppresses telomerase activity in vitro. The integrity of the hTERT–Hsp90–p23 complex is essential for nuclear localization of hTERT, whereas dissociation of p23 results in cytoplasmic retention of hTERT [109]. Another essential Hsp90 client is Tel2, a protein critical for the stability of all six members of the phosphatidylinositol 3-kinase–related kinase (PIKK) family: ATM, ATR, DNA-PKcs, mTOR, SMG1, and TRRAP. These kinases govern key cellular processes, including DNA damage response (ATM, ATR, DNA-PKcs), nutrient sensing (mTOR), nonsense-mediated mRNA decay (SMG1), and transcriptional regulation (TRRAP). Inhibition of Hsp90 disrupts the Tel2–PIKK complex, similar to the effects observed with loss of its co-chaperone Cdc37 [110]. Hsp90 also plays a pivotal role in kinetochore assembly, a dynamic multiprotein structure anchoring chromosome to spindle microtubules during mitosis. The chromatin-binding complex CBF3, composed of Cep3, Ctf13, Skp1, and Ndc10, depends on Hsp90 for maintaining transient interactions between Sgt1 and Skp1/Ctf13, thereby regulating the balance between CBF3 assembly and turnover (Figure 4) [111]. In vitro evidence further indicates that the Hsp90–Sgt1 complex stabilizes the Mis12 complex, which is vital for kinetochore organization [112].

Beyond nuclear functions, Hsp90 contributes to extranuclear protein import, particularly the translocation of preproteins into mitochondria through the translocase of the outer mitochondrial membrane (Tom70) complex (Figure 4). Inhibition of the Hsp90 C-terminal domain (CTD) by novobiocin prevents preprotein complex formation, while GA impairs the generation of translocation intermediates, underscoring Hsp90’s essential role in mitochondrial targeting and import [113].

Moreover, Hsp90 is crucial for the assembly and stability of several macromolecular complexes. A novel ATP-dependent function has been identified in the assembly of the 26S proteasome, where Hsp90 inhibition results in complete disassembly into the 20S core particle and lid component, demonstrating its necessity for proteasomal integrity [114]. In HER2-positive breast cancer cells, proteasome inhibitors induce degradation of ErbB family proteins by triggering Hsp90 inhibition through a chaperone stress response, thereby confirming these proteins as bona fide Hsp90 clients [115]. Similarly, Hsp90 contributes to the assembly of the R2TP complex in *S. cerevisiae*, which regulates the accumulation and processing of box C/D small nucleolar RNAs (snoRNAs). Similarly, in human cells, Rpb1 paralog of the R2TP complex associates with a prefoldin-like complex [116]. Additionally, Hsp90 stabilizes Rpb1 during cytoplasmic assembly of RNA polymerase II [117]. Collectively, these findings highlight Hsp90’s central role in maintaining protein homeostasis through the folding, activation, and assembly of diverse protein complexes. The evidence suggests that combined inhibition of Hsp90 and the proteasome represents a promising therapeutic approach for overcoming drug resistance in cancers.

## 2. Implications of Hsp90 in Health and Diseases

Hsp90 is a highly conserved and abundantly expressed molecular chaperone found in both eukaryotic and prokaryotic cells. It plays a pivotal role in numerous cellular processes, including protein folding, stabilization, degradation, and the regulation of signaling pathways involved in cell proliferation, differentiation, migration, angiogenesis, steroid hormone signaling, organelle-specific stress responses, and apoptosis. There is growing evidence that highlights the significant involvement of Hsp90 in the development of a wide range of human diseases, such as cancer, neurodegenerative disorders, cardiovascular diseases, inflammatory conditions, infectious diseases, and age-related pathologies [118,119]. As a result, several Hsp90 inhibitors are currently in clinical evaluation for the treatment of various malignancies [120,121]. Structurally, Hsp90 consists of distinct domains that allow it to interact with specific client proteins and oncogenic factors. This structural complexity opens up opportunities for developing therapeutic strategies aimed at selectively targeting its binding interfaces. Combining Hsp90 inhibition with client-specific therapeutic agents may represent a promising approach for preventing and treating cancer, as well as other diseases associated with Hsp90.

### 2.1. Hsp90, ER-Localized Grp94, and Mitochondrial Hsp90 in Cancers

Tumor cells often encounter stressful conditions such as heat, nutrient deprivation, hypoxia, and aberrant angiogenesis. In response to these stressors, stress responsive genes are activated, leading to the induced expression of Hsp90 in tumor tissues [122]. The upregulated Hsp90 promotes cancer cell survival, proliferation, and metastasis [123]. Elevated Hsp90 expression has been consistently observed across various types of malignancies. For instance, nearly 90% of primary breast cancers exhibit high levels of Hsp90, which strongly correlate with poor prognosis [124]. Upregulation of Hsp90 has also been reported in other cancers, including multiple myeloma, leukemia, colorectal cancer, non–small cell lung cancer, and prostate cancer [122]. In addition to its intracellular functions, Hsp90 can be secreted by various cancer cell lines and human tumors [77]. Extracellular circulating Hsp90 (eHsp90) has emerged as an early and reliable pan-cancer biomarker for multiple cancers. eHsp90 interacts with cell-surface receptors, including low-density lipoprotein receptor-related protein-1 (LRP1) and toll-like receptors (TLR2 and TLR4), thereby promoting cancer cell proliferation, migration, and invasion [125]. Notably, the co-chaperone of eHsp90, Morgana, which is secreted via unconventional pathways, enhances cancer cell motility via TLR2, TLR4, and LRP1 signaling. Its overexpression in breast cancer has been associated with more aggressive tumor and unfavorable clinical outcomes [126,127]. Therapeutic strategies targeting eHsp90 or co-chaperones like Morgana—such as monoclonal antibodies—have demonstrated promise in suppressing tumor growth by stimulating macrophage-dependent phagocytosis and recruiting CD8+ T cells. These findings highlight novel avenues for anticancer therapy, although further validation is necessary [128].

Hsp90 plays a significant role in multidrug resistance (MDR) in cancer by positively regulating the expression of various resistance-associated proteins, including P-glycoprotein (P-gp/MDR1), breast cancer resistance protein (BCRP), survivin, and Bcl-2 [129,130]. Mechanistically, Hsp90 promotes the accumulation of β-catenin, a key transcriptional regulator of MDR genes, by activating the AKT/GSK3β signaling pathway. In ovarian cancer, this Hsp90-driven activation of β-catenin is a crucial factor underlying resistance to paclitaxel and cisplatin. Inhibiting Hsp90 sensitizes MDR ovarian cancer cells to chemotherapy by impairing the AKT/GSK3β/β-catenin signaling pathway, highlighting Hsp90 as a potential therapeutic target [129]. Additionally, Hsp90 stabilizes several oncogenic signaling proteins. For example, mutant B-Raf, an essential component of the Ras–Raf–ERK signaling cascade that regulates cell proliferation and survival, relies on Hsp90 for its stability and activity (Figure 2) [131]. In breast cancer, estrogen receptor-α (ERα)—a ligand-activated transcription factor that regulates genes associated with cell proliferation—forms a nuclear multiprotein complex with Hsp90 and co-chaperones in the absence of estradiol. Inhibiting Hsp90 promotes the proteasomal degradation of ERα, offering a promising therapeutic strategy in ER-positive breast cancers [123]. Similarly, Hsp90 is crucial for the stability and function of BCR-ABL, a constantly active fusion kinase that drives chronic myeloid leukemia (CML) by activating multiple oncogenic pathways [132]. Additionally, Hsp90 activates NF-κB, a master regulator of inflammation and cell survival, thereby counteracting apoptosis [133].

The tumor suppressor p53, which is mutated in over half of human cancers, plays a central role in apoptosis following DNA damage or oncogenic stress. Multiple studies demonstrated that three main pathways—DNA damage, growth signals (such as those from oncogenes like Ras or Myc), and exposure to chemotherapeutic drugs or ultraviolet light—contribute to the inhibition and degradation of p53, ultimately stabilizing p53 at high levels [134]. Hsp90, in collaboration with Hsp70, binds to mutant p53 and prevents its proteasomal degradation, thereby promoting resistance to apoptosis [135]. Additionally, Hsp90 regulates cell cycle progression by modulating cyclin D-associated kinase activity. Inhibition of Hsp90 disrupts PI3K/AKT signaling (Figure 2) [136], reduces cyclin D-dependent phosphorylation of RB, and induces G1-phase arrest [137]. These findings highlight the potential of Hsp90 inhibitors to target abnormal cell cycle regulation in cancer.

Hsp90 plays a crucial role in cancer cell immortality by supporting the proliferation and survival of cancer cells. It stabilizes telomerase, an enzyme critical for maintaining telomere length, which is essential for the immortality of these cells [138]. The catalytic subunit of telomerase, known as hTERT, interacts with Hsp90 and its co-chaperone p23, forming active telomerase complexes that help preserve telomere length [139]. Additionally, Hsp90 aids in angiogenesis by stabilizing vascular endothelial growth factor receptors (VEGFRs) and fibroblast growth factor receptor 3 (FGFR3) [138]. When Hsp90 is inhibited, these receptors become ubiquitinated and are targeted for degradation, which disrupts angiogenic signaling in tumors [140].

### 2.2. Grp94

Grp94 is essential for maintaining the ER protein-folding capacity, upholding ER stress sensors, and inhibiting ER-associated apoptosis, thus regulating the balance between cancer cell survival and death [141]. As a key mediator of the unfolded protein response (UPR), Grp94 supports plasma cell differentiation, illustrated by the absence of plasma cells in XBP1-deficient systems [142,143]. Additionally, Grp94 stabilizes and protects a wide range of client proteins critical for immune regulation and tumor progression. These include TLRs, the majority of α- and β-integrin subunits, the Wnt co-receptor LRP6, glycoprotein A repetitions predominant (GARP), insulin-like growth factors (IGFs), and the platelet glycoprotein Ib-IX-V complex [144]. Consistent with its functional significance, Grp94 overexpression has been documented in several cancers, including esophageal [145], lung [146], colon [147], gastric [148], and breast carcinomas [149].

Plasma cells, which play a pivotal role in the development of multiple myeloma (MM), rely heavily on the ER for immunoglobulin folding and processing [150]. The excessive accumulation of misfolded proteins leads to ER stress, necessitating robust chaperone activity. To maintain proteostasis, plasma cells depend on ER chaperones such as BiP/Grp78 and Grp94. In vivo studies show that the growth of both XBP1s-transgenic mice and human MM cell lines is dependent on gp96, indicating that inhibiting gp96 could represent a promising therapeutic strategy against MM [143]. Exploiting the unfolded protein response (UPR) has proven effective for treating MM. The combined use of the Hsp90 inhibitor such as, 17-AAG, and the proteasome inhibitor bortezomib induced strong apoptotic responses by activating all three major ER stress sensors [151,152].

Furthermore, Grp94 has also been implicated in the pathogenesis of several solid tumors. In breast cancer, Grp94 is significantly overexpressed compared to normal breast tissues. The genetic ablation of Grp78 and Grp94 in vivo has been shown to reduce tumor proliferation and migration while enhancing apoptosis [153,154]. In triple-negative breast cancer (TNBC), a highly aggressive subtype lacking estrogen receptor (ER), progesterone receptor (PR), and HER2 expression, Grp94 facilitates tumor progression by activating the Wnt/β-catenin pathway, which promotes brain metastasis in TNBC [155]. This adaptation allows cancer cells to survive in hostile microenvironments through the activation of survival-promoting autophagy [156]. Additionally, Grp94 directly stabilizes HER2, a receptor often overexpressed in aggressive breast cancers, thereby amplifying oncogenic signaling cascades that drive tumor growth [157]. Collectively, these findings suggest that dual blockade of Grp94 and autophagy may offer a novel therapeutic strategy for breast cancer.

Grp94 has also been reported to maintain intestinal homeostasis and colon tumorigenesis. Intestinal epithelial cells (IECs) are continuously regenerated by intestinal stem cells, which operate through the canonical Wnt signaling pathway. This regeneration requires Grp94 to work in conjunction with mesoderm development (MesD), an ER chaperone, to ensure the proper surface expression of Wnt co-receptors LRP5 and LRP6 [158]. In a mouse model of colitis-associated colon cancer, a macrophage-specific knockout (KO) of gp96 contributes to colitis and tumorigenesis by enhancing the production of inflammatory cytokines, such as IL-17, IL-23, IL-12, IL-16, and IFN-γ. Mechanistically, Grp94 in macrophages supports inflammation and tumor growth by promoting β-catenin mutations, activating Wnt signaling pathway in colonic epithelial cells, and creating a pro-tumorigenic cytokine environment [159].

### 2.3. Mitochondrial Chaperone TRAP1

Elevated expression of TRAP1 has been reported in various cancers, including hepatocellular carcinoma, breast cancer [160], glioblastoma, small-cell lung cancer [161], renal, prostate, ovarian, pancreatic, and colorectal cancers [160], and esophageal carcinomas. This overexpression is often associated with advanced metastatic tumors and poor prognosis [162]. It has been demonstrated that TRAP1 promotes cell survival by interacting with CypD, a peptidyl-prolyl isomerase that sensitizes the opening of the mPTP. The opening of mPTP, triggered by elevated mitochondrial ROS or Ca^2+^ ions, initiates apoptosis. By binding to CypD, TRAP1 inhibits the opening of PTP, thereby conferring resistance to apoptosis [162]. Additionally, TRAP1 plays a role in the metabolic reprogramming of cancer cells. Despite the availability of oxygen, many tumors rely on glycolysis for rapid ATP generation, a phenomenon known as the Warburg effect [163]. TRAP1 reduces the activity of SDH, which is involved in both the tricarboxylic acid (TCA) cycle and the electron transport chain (ETC). This inhibition leads to the stabilization of hypoxia-inducible factor 1α (HIF1α), promoting tumor growth, although the exact regulatory mechanisms remain incompletely defined [67]. Furthermore, TRAP1 stabilizes and regulates cytochrome c oxidase subunit II (COXII), a critical protein required for ATP synthesis within the ETC. When TRAP1 is inhibited, COXII becomes destabilized, resulting in disruption of the ETC, accumulation of ROS, and apoptosis due to impaired ATP production [164,165,166]. During tumor progression, TRAP1 interacts with NRF2, the master regulator of antioxidant defense, to lower ROS levels [167]. In precancerous cells, it enhances the activity of specific enzymes, like citrate synthase, which fuels the pentose phosphate pathway (PPP) to generate nucleotides and biosynthetic precursors needed for cell proliferation [167]. Moreover, TRAP1 interacts with TBP7, a protein involved in proteostasis and cell cycle regulation, helping to stabilize essential cell cycle regulators such as CDK1 [168]. Downregulation of TRAP1 leads to the degradation of proliferation markers, such as Ki67, CDK1, and MAD2, in breast, colorectal, and lung cancer models, sensitizing these cells to agents that target the cell cycle machinery. These findings suggest that elevated levels of TRAP1 may contribute to resistance against therapies aimed at disrupting cell cycle processes [70].

Overall, these findings underscore Hsp90’s role as a central regulator of various processes, including multidrug resistance, oncogenic signaling, evasion of apoptosis, cell cycle progression, telomerase activity, and angiogenesis. This makes Hsp90 an attractive therapeutic target for treating a wide range of malignancies.

### 2.4. Clinical Applications of Hsp90 Inhibitors in Cancer

Hsp90 inhibitors have emerged as promising agents for cancer treatment due to their ability to destabilize a wide range of oncogenic client proteins. Researchers have explored both natural products and synthetic derivatives in preclinical and clinical settings.

### 2.5. Curcumin-Based Hsp90 Inhibitors

Curcumin derivatives have shown potential as novel Hsp90 inhibitors. The compound, 4-(4-pyridinylmethylene) curcumin (C1206), suppresses the ATPase activity of Hsp90, leading to the inhibition of chronic myeloid leukemia (CML) cell proliferation [169]. Similarly, another derivative, C0818, binds to the C-terminal dimerization domain of Hsp90 and inhibits its ATPase activity as well [170]. Additionally, the compound (E)-2-(4-hydroxy-3-methoxybenzylidene)-5-(E)-3-(4-hydroxy-3-methoxyphenyl) acryloyl) cyclopentanone (CUR3d) downregulates the expression of both Hsp90AA1 and Hsp90AB1. This results in growth inhibition, increased cytotoxicity, activation of caspase-3, and DNA fragmentation in hepatocellular carcinoma (HCC) cells [171]. Moreover, combination therapy that incorporates curcumin along with histone deacetylase (HDAC) inhibitors like vorinostat and panobinostat has proven effective. This combination enhances the depletion of Hsp90 client proteins, producing a synergistic effect that leads to increased growth inhibition in squamous cell carcinoma (SCC) A431 and mesothelioma STO cells [172].

### 2.6. Geldanamycin and Derivatives

Geldanamycin is a natural benzoquinone ansamycin that binds to the N-terminal ATP-binding pocket of Hsp90. This interaction inhibits the chaperone function of Hsp90 and disrupts cell proliferation. However, the clinical development of geldanamycin was limited due to issues such as hepatotoxicity, metabolic instability, and poor solubility. As a result, researchers developed less toxic derivatives, including 17-allylamino-17-demethoxygeldanamycin (17-AAG) and 17-dimethylaminoethylamino-17-demethoxygeldanamycin (17-DMAG), which showed improved potency and tolerability [173]. 17-AAG retains potent anticancer activity while reducing hepatotoxicity and improving bioavailability [174]. It binds to the ATP-binding site of Hsp90, inhibiting the formation of multi-chaperone complexes and promoting the ubiquitin-proteasome-mediated degradation of client proteins [175]. In cases of HER2-positive breast cancer, combining trastuzumab with 17-AAG resulted in clinical benefits for 59% of patients. In neuroblastoma with the ALK gene mutation F1174L, which confers resistance to crizotinib, the combination of 17-AAG with the ALK inhibitor TAE684 restored sensitivity to treatment [176]. In thyroid cancer, 17-AAG induced the depletion of Raf-1 and AKT, which inhibited MEK1/2 phosphorylation, suppressed cell signaling, and reduced cell proliferation [177,178]. Additionally, in leukemia, 17-AAG facilitated the shift of BCR-ABL from Hsp90 to Hsp70, promoting its proteasomal degradation [132]. It also decreased Bcl-2 expression in acute promyelocytic leukemia (APL), triggering apoptosis. These findings underscore the potential of 17-AAG in combination therapies for both hematological malignancies and solid tumors [179].

17-DMAG, a water-soluble analog of 17-AAG, binds to the ATP-binding site of Hsp90, resulting in the misfolding and subsequent proteasomal degradation of client proteins [180]. In acute myeloid leukemia (AML), 17-DMAG decreased the expression levels of HIF-1 and VEGF, thereby inhibiting angiogenesis and tumor growth [181]. In case of metastatic pancreatic carcinoma and lung cancer, 17-DMAG exhibited superior efficacy compared to 17-AAG [182]. In breast and ovarian cancers, 17-DMAG efficiently targeted HER2, leading to its degradation [183]. In HCC, 17-DMAG promotes the degradation of Survivin, inhibits NF-κB, increases p53 levels, and reduces Cyclin D1 expression, collectively suppressing cell proliferation [184]. Phase I clinical trials have explored the use of 17-DMAG both as a monotherapy and in combination regimens for various hematological and solid malignancies. However, further validation in phase II and III trials is still needed to confirm the specificity and efficacy (Table 2) [185].

### 2.7. Purine-Based Hsp90 Inhibitors

Purine-based Hsp90 inhibitors have been extensively studied for their ability to target Hsp90 both in vitro and in vivo across various cancer cell lines and models. Zelavespib (PU-H71) has shown significant activity in several tumor types, including lymphoma, metastatic solid tumors, and myeloproliferative neoplasms (MPNs). Recently, it was evaluated in relation to acute myeloid leukemia (AML) that carries the PML–SYK fusion [186]. CUDC-305 has demonstrated potent antitumor effects in diverse preclinical models, including NSCLC, glioblastoma, triple-negative breast cancer, AML, and colorectal cancer. In an H1975 NSCLC xenograft model, CUDC-305 inhibited subcutaneous tumor growth and promoted degradation of multiple Hsp90 client proteins, such as mutant EGFR and key regulators of the RAF/MEK/ERK and PI3K/AKT signaling pathways (Figure 2) [187]. BIIB021 and BIIB028, both of which are orally bioavailable Hsp90 inhibitors, were developed for the treatment of advanced solid tumors (Table 2) [188].

### 2.8. Small Molecule Inhibitors of Hsp90

Newer generations of Hsp90 inhibitors include small molecules that target distinct domains. SL-145, a C-terminal inhibitor, has demonstrated potent antitumor and anti-metastatic activity without triggering the heat shock response in TNBC cells. This is achieved by suppressing the AKT, MEK/ERK, and JAK2/STAT3 signaling pathways both in vitro and in vivo [189]. Another innovative compound, Gamitrinib, a mitochondrial-targeted Hsp90 inhibitor, along with its derivatives such as G-G4, selectively disrupts Hsp90 and CypD in mitochondria. These compounds exhibit strong antitumor efficacy in multiple xenograft models, including the Transgenic Adenocarcinoma of the Mouse Prostate (TRAMP) model, by promoting the degradation of mitochondrial Hsp90 and CypD [190]. Epigallocatechin-3-gallate (EGCG) is another Hsp90 inhibitor that binds to the CTD and disrupts the activity of client proteins, such as telomerase and aryl hydrocarbon receptor (AhR) [191]. Similarly, HTS, also known as 3-propylpyrimido (5,4-e) (1,2,4) triazine-5,7-dione (C9), disrupts client binding and reduces Hsp90 activity [191]. Additionally, Sansalvamide A-amide (San A-amide) serves as a small molecule inhibitor of Hsp90, binding between the NTD and the MD to disrupt their functions in various cancers, including pancreatic, colon, breast, and prostate cancers, both in vivo and in vitro [192]. Ganetespib, which contains a resorcinol ring, is another Hsp90 inhibitor with reduced cardiac and liver toxicity. It exhibits robust cellular potency against drug-resistant tumors in various human malignancies [193]. SNX-2112 is an ATP-competitive Hsp90 inhibitor that has demonstrated antitumor activity in both hematological and solid tumor malignancies [194]. Pimitespib is an orally active Hsp90 inhibitor that selectively targets Hsp90α/β, while exhibiting minimal affinity for Grp94 and TRAP1. In Gastrointestinal stromal tumors (GIST), it demonstrated strong antitumor activity and has been approved for the treatment of GIST patients in Japan. Furthermore, combination therapy of pimitespib with the anti-PD-1 antibody nivolumab showed an acceptable safety profile and promising antitumor activity (ORR 16%) in patients with advanced solid tumors, particularly [195]. Furthermore, novel orally bioavailable ATP-competitive inhibitors, such as 2-aminothieno [2,3-d] pyrimidine and NVP-BEP800, have shown oral efficacy in animal models of cancer [196]. A list of these inhibitors is presented in Table 2. In addition to those mentioned, many other inhibitors have been demonstrated in various cancer cell lines and in vivo models, though they may be beyond the scope of this review.

**Table 2:** Hsp90 inhibitors, their mechanism of action, and therapeutic role in cancer.

S. No.	Hsp90 Inhibitors	Derivatives	Binding Sites	Clinical Phase/Trails	Action Mechanism	Diseases	Reference
1.	Curcumin and derivatives	C212	Reduces Hsp90 level	Preclinical	Targets client protein	Leukemia cells	[197]
		C1206	MD	In vivo/in vitro	Inhibits ATPase activity	CML	[169]
		C0818	CTD	In vivo/in vitro	Inhibits ATP hydrolysis	In vivo and in vitro tumor models	[170]
		CUR3d	Reduces Hsp90 expression	Phase I	Modulates multiple signaling pathways and client proteins	HepG2, hepatocellular cancer	[171]
2.	Geldanamycin and derivatives	17-AAG	NTD	Phase III	Inhibits ATPase activity	Breast, Leukemia, Lung; thyroid cancer	[177,179]
		17-DMAG	NTD	Phase I	Targets client proteins	HCC, HepG2, AML	[180,181]
		IPI-504	NTD	Phase III	Inhibits ATPase activity	GIST	[198]
		IPI493	Impairs signaling protein kinases	Phase III	Acts as tyrosine kinase inhibitor	GIST	[199]
3.	Purine-based inhibitors	CUDC-305	NTD	Phase I	Tyrosine kinase inhibition	Breast cancer, NSCLC	[187]
		PU-H71	NTD	Phase I	Targets client proteins	TNBC, myeloma; HCC	[200]
		PU3	NTD	In vivo/in vitro	Cell cycle inhibition	Breast cancer	[201]
		MPC-3100	NTD	Phase I	Inhibits apoptosis	Breast cancer	[202]
		BIIB021	NTD	Phase II	Inhibits ATPase activity	Xenograft models	[188]
4.	Small-molecule inhibitors/benzamide-scaffold inhibitors	Novobiocin (KU135; A4)	CTD	Phase I	Disrupts the dimerization and promotes release of client proteins	Breast cancer	[203]
		Epigallocatechin gallate (EGCG)	CTD	Phase IV	Targets XAP2-bound Hsp90 complex	Pancreatic, prostate cancer	[191,204]
		SNX-2112, SNX-5422	NTD	Phase I	Induces cell cycle arrest; inhibits eNOS and AKT signaling	NSCLC, SCLC, multiple myeloma	[194]
		AT13387 (Onalespib)	NTD	Phase I/II	Depletes Hsp90 client proteins	Prostate cancer	[205]
5.	Tryptophan (TRP) analogs	XL888	NTD	Phase I	Inhibits cell cycle; targets client proteins	Colorectal, pancreatic cancer	[206]
		San A-amide	N-middle domain	Phase I/II	Allosterically disrupts CTD protein binding	Pancreatic, breast, colon cancers	[192]
6.	Resorcinol-based inhibitors	NVP-AUY922 (Luminespib)	NTD	Phase I/II	Inactivates apoptotic pathways	Multiple myeloma, solid tumors	[207]
		Ganetespib	N-terminal ATP-binding site	Phase I/II	Induces cell cycle arrest and apoptosis	Ovarian, breast cancer	[193]
		Pimitespib (TAS-116)	NTD	Phase III	Inhibits growth and induces apoptosis	GIST, colorectal cancer	[195]

## 3. Hsp90 in Alzheimer’s Disease (AD) and Neurodegeneration

Hsp90 has emerged as a critical regulator in the pathogenesis of multiple neurodegenerative disorders, including AD, Parkinson’s disease (PD), and Huntington’s disease (HD). Numerous studies have demonstrated that Hsp90 provides neuroprotective effects by maintaining protein homeostasis and preventing the accumulation of misfolded proteins. Specifically, Hsp90 has been found to reduce amyloid-β (Aβ) aggregation and plaque formation. Further, extracellular Hsp90 influences immune responses by activating phagocytes via the Toll-like receptor 4 (TLR4) pathway, which helps promote Aβ clearance. Hsp90 also plays a role in the phosphorylation and dephosphorylation of tau by stabilizing tau kinases [208]. Despite this, several co-chaperones, such as protein phosphatase 5 (PP5), Cdc37, and CacyBP/SIP, are essential for tau dephosphorylation [209]. CacyBP/SIP, a Siah-interacting protein that is highly expressed in the brain, is predominantly localized to neuronal somata in AD patients compared to healthy controls. It associates with phosphorylated tau and β-tubulin and protects against α-synuclein aggregation in Lewy bodies within the substantia nigra, indicating its potential involvement in both AD and PD pathologies. Modulating Hsp90 activity may affect CacyBP/SIP function and, consequently, influence tau phosphorylation and α-synuclein aggregation. However, further validation through in vitro and in vivo studies is needed.

Cdc37 is a co-chaperone that consists of three domains: NTD, MD, and CTD. It interacts with Hsp90 through its stable middle region [210]. Cdc37 plays a crucial role in stabilizing tau kinases, including Cdk5 and Akt, and enhances their activity by facilitating interactions with Hsp90. Research has demonstrated that inhibiting Hsp90 not only helps clear tau but also regulates the activation of the tau kinase Cdk5 [211]. In AD, the abnormal hyperactivation of Cdk5 is known to contribute to tau pathology. Therefore, Hsp90 inhibition promotes tau clearance while also modulating Cdk5 activity, which reduces neurotoxic kinase signaling [212]. Disrupting the Hsp90–Cdc37 complex using small molecules, such as celastrol, withaferin A, platycodin D, and kongensin A, either individually or in combination with 17-AAG, can destabilize neurotoxic kinase assemblies. This offers a potential therapeutic strategy for treating neurodegenerative diseases [213].

The E3 ubiquitin ligase, carboxyl terminus of the Hsp70-interacting protein (CHIP) plays a critical role in the degradation of misfolded or aggregated tau proteins through the ubiquitin–proteasome system (UPS). Reduced levels of CHIP lead to the accumulation of pathological tau, and impairment of the co-chaperone STI1/Hop has been implicated in tauopathies. CHIP forms functional complexes with Hsp70, Hsp90, and tau, positioning Hop as a key regulator that facilitates tau clearance via the UPS [214]. These observations suggest that age-related decline in co-chaperone function may contribute to the development of neurodegenerative disorders, such as AD [215]. However, under pathological conditions, Hsp90 activity paradoxically promotes tau aggregation, which makes Hsp90 inhibition a promising strategy for reducing tau accumulation and mitigating amyloid-beta (Aβ) toxicity. Hsp90 inhibitors are generally classified into two categories: N-terminal and C-terminal inhibitors. N-terminal inhibitors such as geldanamycin and its less toxic derivatives, 17-AAG and 17-DMAG disrupt ATP binding, thereby promoting tau clearance [216]. Additionally, purine-based N-terminal inhibitors, such as EC102 and PU24FCI, have demonstrated effectiveness in reducing tau levels [211]. C-terminal inhibitors, including celastrol, novobiocin, and the derivatives KU-32 and A4, exert neuroprotective effects by modulating client protein interactions without inducing the heat shock response. These agents have shown potential in models of mitochondrial dysfunction and diabetic peripheral neuropathy in an Hsp70-dependent manner [217]. For instance, in the Tg2576 mouse model and cultured neurons, 17-AAG mitigated damage from soluble Aβ and enhanced synaptic protein expression through the activation of heat shock factor 1 (HSF1) [218]. Similarly, radicicol exhibits comparable neuroprotective effects and is considered a promising candidate for the treatment of neurodegenerative diseases [219].

Hsp90 co-chaperone complexes play dynamic and critical roles in tau metabolism, significantly impacting the progression of neurodegenerative disease. Various co-chaperones, such as ATPase homolog 1 (Aha1), Cdc37, and peptidyl-prolyl cis-trans isomerases FKBP51/52, along with other PPIase complexes, and the Hsp-organizing protein (Hop), are often overexpressed with aging and contribute to disease development. For instance, the overexpression of Aha1 accelerates tau fibrillization, while FKBP52 interferes with AMPA receptor binding, which impairs synaptic plasticity [83,219]. Pharmacological inhibition of Aha1 using KU-177 has been shown to reduce tau toxicity in models of AD. Additionally, elevated levels of FKBP51 correlate with increased tau oligomer accumulation. Given its strong affinity for Hsp90, targeting FKBP51 could represent a viable strategy to modulate tau phosphorylation and the pathology of AD [220].

Similarly, overexpression of HOP exacerbates α-synuclein toxicity in PD and amyloid toxicity in AD [221]. On the other hand, knocking down Hop facilitates tau accumulation, indicating that both Hsp70 and Hsp90 work together to chaperone tau, each relying on the other for efficient clearance [222]. Inhibition of cytosolic Hsp90 triggers a compensatory upregulation of Hsp70, which may confer neuroprotection. For example, 17-AAG has been shown to improve synaptic function in AD mouse models [218], while SNX-0723 reduces α-synuclein toxicity in PD rat models [223,224]. Targeting Hsp90 co-chaperones may enhance therapeutic specificity by selectively targeting tau and minimizing off-target effects. Although several pan-Hsp90 inhibitors have reached phase II clinical trials, their application is limited due to dose-dependent hepatotoxicity and ocular toxicity resulting from non-selective isoform inhibition [225]. Therefore, developing isoform-selective or co-chaperone-directed modulators represents a promising approach to harness Hsp90 biology therapeutically while reducing adverse effects in neurodegenerative diseases.

## 4. Hsp90 in Cardiac Disease and Diabetes

Hsp90 is essential for the proper functioning of eukaryotic cells, as it helps maintain proteostasis and supports cellular viability. However, aging is associated with a decline in Hsp90 levels, which can lead to proteotoxic stress, accelerated cellular senescence, and disrupted protein homeostasis. Experimental evidence has shown that cells need approximately 50–70% of normal Hsp90 levels to remain viable; levels below this threshold result in senescence, collapse of proteostasis, and ultimately cell death [226]. Hsp90, along with its co-chaperones and client proteins, is involved in signaling pathways linked to cardiac diseases, such as MAPK, PI3K/AKT/mTOR, and TNF-α signaling (Figure 2) [227]. In metabolic disorders like cardiovascular disease and diabetes, the chaperone functions of Hsp90 and its interactions with client proteins become particularly important, as cells face increased stress and pathological changes. Hsp90 plays a crucial role in signaling pathways associated with cardiomyopathy. Specifically, the expression of Hsp90α is elevated in cardiac tissues from patients with obstructive hypertrophic cardiomyopathy (HCM) and in mice subjected to transverse aortic constriction (TAC). The increased levels of secreted Hsp90α (eHsp90α) are linked to chronic pressure overload, which triggers cardiac hypertrophy and dysfunction. Research has indicated that pharmacological or genetic removal of Hsp90α or eHsp90α can reduce cardiac hypertrophy by inhibiting β-catenin/TCF7 signaling through N-cadherin-mediated pathways under pressure overload conditions. These findings suggest that the Hsp90α/N-cadherin axis could be a potential therapeutic target for regulating cardiac remodeling in pressure-overload situations and other cardiovascular diseases [228].

TGF-β-Smad2/3 signaling plays a crucial role in fibroblast-mediated cardiac fibrosis, myocardial infarction, and ischemia/reperfusion (I/R) injury [229]. Hsp90 is responsible for stabilizing Smad1/5 and facilitating their movement into the nucleus, thereby functioning downstream of both canonical and non-canonical TGF-β pathways [230]. When Smad2/3 or Tgfbr1/2 is knocked down in cardiac fibroblasts, there is a decrease in fibrosis and extracellular matrix remodeling. Notably, the knockdown of Tgfbr1/2 specifically alters the expression of genes critical to maintaining cardiomyocyte homeostasis and adaptive responses. This suggests that TGF-β–Smad2/3 signaling in activated fibroblasts is a central mediator of the fibrotic response [229]. Additionally, Hsp90 stabilizes various kinases and transcription factors that contribute to cardiac hypertrophy, inflammation, and fibrosis. For instance, Hsp90 stabilizes the IκB kinase (IKK) complex, which is a key regulator of NF-κB signaling. Inhibition of Hsp90 using geldanamycin disrupts TNFα-induced NF-κB activation in cardiomyocytes [231,232]. The activation of NF-κB leads to the expression of pro-inflammatory cytokines that contribute to hypertrophy, fibrosis, and tissue repair following I/R injury. Furthermore, Hsp90AA1 serves as a target of miR-1 in the context of I/R injury. In vivo, miR-1 negatively regulates the expression of Hsp90AA1, while restoring Hsp90AA1 levels helps to reduce cardiac damage induced by I/R [233]. Hsp90 also regulates the differentiation of fibroblasts into myofibroblasts (MF) and adventitial remodeling through calcineurin (CN) and dynamin-related protein 1 (Drp1) in response to angiotensin II (AngII).

NF-κB plays a role in AngII-induced cardiac hypertrophy through interactions with Hsp90. Inhibiting Hsp90 with geldanamycin prevents cardiac hypertrophy by lowering IKKα/β levels and blocking NF-κB activation [234]. In metabolic diseases like diabetes, cytosolic Hsp90 has complex regulatory functions. It is upregulated in diabetic tissues as part of the cellular stress response and interacts with key metabolic signaling proteins [235]. Notably, Hsp90 binds to components of the insulin signaling pathway. When Hsp90 is pharmacologically inhibited with AUY922 in insulin-resistant cells, JNK activation decreases, insulin signaling improves, and that mimics the beneficial effects of elevated Hsp70 [235,236,237]. Chronic treatment with Hsp90 inhibitors in obese or diabetic mouse models activates the Hsf1–Hsp70 stress pathway, leading to improved glucose control, reversal of hyperglycemia, and enhanced insulin sensitivity [235,238]. These findings highlight Hsp90 as a central regulator of cardiac fibrosis, hypertrophy, inflammation, and metabolic dysfunction, suggesting that targeted modulation of Hsp90 could be a promising therapeutic strategy for cardiovascular and metabolic diseases.

### 4.1. Grp94 in Metabolic Disease and Cardiac Hypertrophy

**Figure 5:**
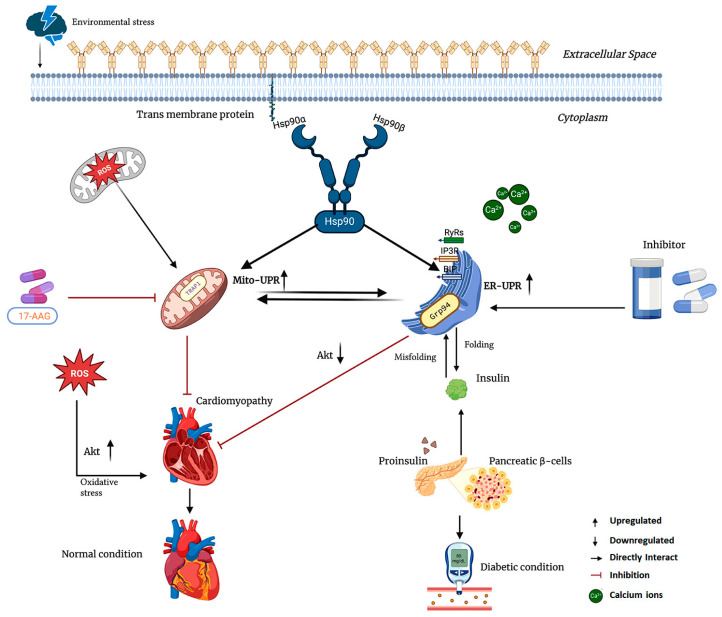
A schematic illustration of roles of the ER and mitochondria in cardiomyopathy and diabetes, and potential therapeutic strategies. The schematic illustrates how ER and mitochondria act as primary targets under diverse cellular stress conditions. Pharmacological inhibition of Hsp90 and its client proteins with agents such as 17-AAG, 17-DMAG, and novobiocin can disrupt ER stress responses and client protein degradation, affecting pathways including reactive oxygen species (ROS) regulation, oxidative phosphorylation (OXPHOS), and the unfolded protein responses (UPRs). Small-molecule inhibitors, particularly natural compounds, and antioxidants, may help alleviate cellular dysfunctions associated with cardiomyopathy and diabetes, especially in the early stages of these diseases.

Grp94, also known as gp96, is a chaperone located in the ER that is essential for the proper folding and quality control of secreted and membrane proteins. Its importance in metabolic diseases is highlighted by its role in insulin production. Pancreatic β cells depend on Grp94 for the correct folding of proinsulin within the ER. Grp94 physically associates with proinsulin, and any inhibition or knockdown of Grp94 leads to misfolded insulin and a reduction in glucose-stimulated insulin secretion [239,240]. Experimental models have shown that inhibiting Grp94 shortens the half-life of proinsulin, decreases intracellular insulin levels, and triggers mild endoplasmic reticulum stress through PERK signaling in β cells (Figure 5) [240,241]. In type 2 diabetes, β cells upregulate Grp94 mRNA as an adaptive response, underscoring its critical role in maintaining protein balance during periods of high secretory demand. Thus, adequate Grp94 activity is vital to protecting β cells from failure by ensuring efficient management of insulin.

Grp94 plays a crucial role in the heart by contributing to the unfolded protein response and supporting the survival of cardiomyocytes during stress. When Grp94 is overexpressed in cardiomyocytes, it helps reduce cell death in situations such as calcium overload or simulated ischemia [241,242,243]. By acting as a chaperone for calcium-handling proteins and ER stress sensors, Grp94 protects cardiac muscle cells from injury. In cases of diabetic cardiomyopathy, chronic high blood sugar levels lead to ER stress, which can deplete adaptive chaperones like Grp94. Research on human hearts affected by diabetes has shown a notable decrease in Grp94 expression, indicating that ER dysfunction may increase cardiac vulnerability in these patients [244,245]. Conversely, studies have found that alleviating ER stress in diabetic rodents can restore Grp94 levels and improve cardiac function. In summary, Grp94 is a key mediator of proteostasis in both pancreatic beta cells and cardiomyocytes. By maintaining the protein-folding capacity of the ER, Grp94 serves as a protective factor under metabolic stress. Its dysfunction can lead to impaired insulin secretion and heightened susceptibility of the heart to injury.

### 4.2. Hsp90 Inhibitors and Their Role in Cardiac Disorder and Diabetes

Curcumin is a natural polyphenol derived from *Curcuma longa*, known for its potent anti-inflammatory and antioxidant properties. Numerous studies have shown that curcumin interacts with Hsp90, hindering its chaperone function and leading to the depletion of client proteins. While its effectiveness as a direct Hsp90 inhibitor is relatively modest, this interaction provides a mechanistic basis for some of its anti-inflammatory and metabolic effects [172]. In models of metabolic disease, curcumin has been found to improve glucose tolerance and enhance insulin sensitivity in obese and diabetic rodents. These benefits are attributed to increased Akt signaling, regulation of sterol regulatory element-binding proteins (SREBPs), and a reduction in inflammation and oxidative stress through the suppression of NF-κB and c-Jun N-terminal kinase (JNK) pathways (Figure 5) [246]. Additionally, in certain models, the metabolic benefits of curcumin are mediated through gut microbiota-dependent induction of fibroblast growth factor 15 (FGF15) signaling [247,248]. In summary, these findings support a systems-level mechanism in which partial modulation of Hsp90 activity, combined with the regulation of multiple pathways, contributes to the cardiometabolic advantages of curcumin.

### 4.3. Geldanamycin (GA) and Its Derivatives Role in Cardiac Disorders and Diabetes

Geldanamycin (GA) and its analogs represent a class of Hsp90 inhibitors that target Hsp90 by binding to its N-terminal ATP-binding pocket, disrupting its chaperoning functions. Studies have demonstrated that using GA or inhibitory peptides can attenuate profibrotic TGF-β signaling in both cardiomyocytes and cardiac fibroblasts [249]. However, the clinical use of GA has been limited due to dose-dependent hepatotoxicity, which is linked to the benzoquinone component of its structure. To address this issue, researchers have synthesized many low toxicity derivatives by modifying the quinone ring structure, including 17-AAG, 17-DMAG, and novobiocin. These analogs display improved pharmacological profiles with reduced toxicity, enhanced metabolic stability, and better tolerability [250].

In experimental models of cardiac disease, administering 17-AAG after myocardial infarction or pressure overload has been shown to reduce pathological remodeling. This effect is partly mediated by the accelerated degradation of Raf-1 and the subsequent attenuation of ERK/GATA4 signaling. Additionally, Hsp90 inhibition has been shown to suppress necroptotic signaling pathways (RIP1/RIP3/MLKL) and NF-κB activation, which helps to reduce cardiomyocyte death [227,251]. In a transverse aortic constriction (TAC) model, treatment with 17-AAG resulted in the downregulation of the Hsp90 client protein calcineurin and the transcription factor NFATc2 in cardiac fibroblasts, further supporting its anti-remodeling effects [252].

Similarly, 17-DMAG has demonstrated protective effects in vascular disease models. In case of atherosclerosis, 17-DMAG reduced ERK phosphorylation and suppressed oxidative stress [253]. Inhibition of Hsp90 decreases oxidative effects by disrupting its interaction with Nox1 [254]. In vivo, treatment with 17-DMAG in diabetic mice attenuated the progression of atherosclerosis. This effect was associated with enhanced autophagy, increased expression of antioxidant enzymes (such as catalase, superoxide dismutase, and heme oxygenase-1), and induction of Hsp70 [255]. In apolipoprotein E-deficient diabetic mice, 17-DMAG reduced atherosclerotic plaque burden by approximately 60% and promoted the development of a more stable plaque phenotype. These vascular benefits were linked to the suppression of NF-κB/STAT signaling and the activation of the Nrf2 antioxidant pathway, occurring independently of changes in glucose or lipid levels [256,257]. In the context of metabolic disease, selective Hsp90 inhibition using the synthetic inhibitor AUY922 has shown positive effects. AUY922 suppressed JNK1 phosphorylation, activated the Hsf1–Hsp70 cytoprotective axis, and improved insulin signaling in vitro. Chronic treatment with AUY922 in db/db homozygous mice reversed hyperglycemia, while in diet-induced obese mice, it enhanced insulin sensitivity [235,258]. Across multiple models of diabetic complications—including nephropathy, neuropathy, and atherosclerosis—Hsp90 inhibition consistently alleviated pathology by dampening inflammatory and stress-related signaling pathways.

### 4.4. Mitochondria-Directed and Extracellular Hsp90 Inhibition

Mitochondrial Hsp90 (mtHsp90), also referred to as TRAP1, has emerged as a promising therapeutic target in cardiopulmonary disease. Gamitrinib, a mitochondria-targeted Hsp90 inhibitor, selectively disrupts the function of mtHsp90. In models of pulmonary arterial hypertension (PAH), Gamitrinib has been shown to reduce the viability and proliferation of pulmonary artery smooth muscle cells (PASMCs) [259]. Inhibition of mtHsp90 with Gamitrinib effectively reversed pulmonary vascular remodeling and improved cardiac output in preclinical PAH models, with no evidence of systemic toxicity. Extracellular Hsp90 has also been implicated in cardiac pathology. Celastrol, a bioactive triterpenoid compound derived from Tripterygium wilfordii and Celastrus orbiculatus (both from the Celastraceae family), inhibits Hsp90 by blocking its interaction with CDC37 at the C-terminal domain. This action disrupts the Hsp90–CDC37 complex [213]. Celastrol exerts cardioprotective effects by inducing cytoprotective proteins such as Hsp70 and heme oxygenase-1 (HO-1). In ischemia–reperfusion (IR) injury models, Celastrol, when administered either as a pre-treatment or at the time of reperfusion, significantly reduced cardiac injury. This protective effect was associated with the suppression of STAT3 activation in angiotensin II-challenged mice, enhanced cardiomyocyte viability, and reduced mPTP opening, thereby preventing cardiac cell death [260].

## 5. Conclusions and Future Direction

HSPs can be considered the cellular “engines,” much like an engine drives a vehicle; without their proper function, cells are unable to maintain normal physiology. Hsps orchestrate diverse cellular activities, including protein folding and refolding of denatured or misfolded proteins [261], cell cycle regulation, survival pathways, protein homeostasis, and hormone and signal transduction [262]. As a result, they are essential for maintaining cellular homeostasis, and dysfunction in these proteins has been linked to a wide range of human diseases. Among the Hsp family, Hsp90 functions as a central molecular chaperone, orchestrating the stability, maturation, and activity of numerous client proteins with the help of co-chaperones. The repertoire of Hsp90 client proteins continues to expand, suggesting that additional client interactions remain to be identified. Beyond its canonical role in protein folding, Hsp90 has been extensively characterized as a key regulator in human diseases, particularly cancer, as well as in neurodegenerative disorders and metabolic pathologies such as cardiac dysfunction and diabetes. Since Hsp90 is vital for the maturation and stabilization of numerous oncogenic kinases, pharmacological inhibition of Hsp90 disrupts these clients, leading to their degradation. Despite the advancements in understanding Hsp90, several critical questions remain unanswered. It is still unclear how Hsp90 recognizes such a diverse array of client proteins and how co-chaperones refine client specificity and activity. Moreover, the relative contributions of distinct Hsp90 isoforms, including those localized to the ER and mitochondria, to client protein maturation are not fully understood. Elucidating these mechanisms will be crucial for the rational design of more selective Hsp90-targeted therapies for cancer and other human diseases.

Despite significant advances in our understanding of Hsp90 biology, several challenges remain. The molecular principles that govern Hsp90’s recognition of structurally diverse clients, the fine-tuning of specificity by co-chaperones, and the distinct roles of its isoforms—particularly those localized in the ER and mitochondria—are not yet fully elucidated. These gaps limit the rational development of selective Hsp90-targeted therapeutics. Inhibition of Hsp90 leads to destabilization of client proteins, which then undergo ubiquitination and are subsequently degraded by proteasomes [131]. To date, no Hsp90 inhibitor has achieved full clinical approval, primarily due to issues related to toxicity and compensatory cellular responses, such as the activation of Hsf1, leading to the subsequent upregulation of other Hsps. Emerging strategies are being developed to overcome these limitations, including the following:

- Designing small molecules or peptide analogs of the TRP domain that disrupt Hsp90 complex assembly by competitively binding the CTD.
- Targeting PTMs that regulate Hsp90-client or Hsp90–co-chaperone interactions, either genetically or pharmacologically, to enhance the effectiveness of existing inhibitors.
- Co-inhibiting HSF1 to prevent stress-induced feedback responses that attenuate the effects of Hsp90 blockade.
- Developing isoform-specific inhibitors, particularly targeting Hsp90α, which has been associated with cardiac hypertrophy and heart failure due to pressure overload.
- Integration of organelle-specific targeting motifs into pan-Hsp90 inhibitors to promote their selective accumulation in target organelles, including mitochondria and the ER.

Additionally, PTMs that influence Hsp90’s interaction networks play a crucial role in cardiomyopathies and other pathologies. Small molecules designed to modulate these modifications may offer a promising therapeutic avenue. A deeper mechanistic understanding of Hsp90’s structural dynamics, client specificity, isoform biology, and regulatory PTMs will be essential for the development of next-generation therapies. Further, mutational studies that induce conformational changes in Hsp90, disrupting assemblies such as epichaperome without impairing its folding capability, could provide valuable insights. Integrating these insights with innovative inhibitor design could enable precise targeting of Hsp90 across a range of conditions, including malignancies, neurodegenerative disorders, cardiovascular diseases, and diabetes, thereby maximizing therapeutic efficacy while minimizing adverse effects.

## Figures and Tables

**Table 1:** Hsp90 isoforms, main characteristics, and biological functions.

Localization	Gene/Protein	Characteristics	Homology	Functions	References
Cytosol	Hsp90α (Hsp90AA1)	Stress inducible	~86% amino acid sequence similarity between Hsp90α and Hsp90β; >95% homology within N-terminal and ATP-binding domains	Chaperoning and maturation of client proteins; regulation of steroid hormone receptor signaling; facilitation of protein trafficking; cell-cycle control	[2]
	Hsp90β (Hsp90 AB1)	Constitutively expressed			
Endoplasmic reticulum (ER)	Grp94/Gp96 (Hsp90B1)	Ca^2+^-binding chaperone; ATP hydrolysis triggers conformational activation	>50% amino acid sequence homology with cytosolic Hsp90; possesses a KDEL retention motif; lacks MEEVD sequence	Folding and maturation of secretory and membrane proteins; quality control of ER clients such as TLR, insulin-like growth factors	[5]
Mitochondria	TRAP1 (TNF receptor-associated protein 1)	Cytoplasmic and imported into mitochondria; mitochondrial targeting sequence cleaved upon import	>85% identity with N-terminal ATP-binding domain of cytosolic Hsp90; lacks charged linker region and MEEVD motif	Maintains mitochondrial integrity; protects against ROS and oxidative stress; overexpressed in several cancers	[6,7,8]
Extracellular medium	eHsp90α (plasma and serum); eHsp90β (exosome secretion dependent on p53 activation in cancer cells)	Cell surface-associated and secreted forms of Hsp90	High homology with cytosolic Hsp90; contains a distinct 115-amino-acid middle and charged linker binding domain	Facilitates tumor cell invasion, migration, and extracellular matrix remodeling	[9,10]

## Data Availability

No new data were created or analyzed in this study. Data sharing is not applicable to this article.

## Abbreviations

The following abbreviations are used in this manuscript:

17-AAG	17-allylamino-17-demethoxygeldanamycin
17DMAG	17-dimethylaminoethylamino-17-demethoxygeldanamycin
Aβ	Amyloid-β
AD	Alzheimer’s disease
AHA1	Activator of Hsp90 ATPase homolog 1
AML	Acute Myeloid Leukemia
AGO	Argonaute
Ask1	Apoptosis signal–regulating kinase 1
CTD	C-terminal dimerization domain
COXII	Cytochrome c oxidase subunit II
ER	Endoplasmic Reticulum
eHsp90	Extracellular Hsp90
FGFR3	Fibroblast growth factor receptor 3
GARP	Glycoprotein A repetitions predominant
GIST	Gastrointestinal stromal tumors
GR	Glucocorticoid receptor
Grp94	Glucose-regulated protein 94
HCC	Hepatocellular carcinoma
HCM	Hypertrophic cardiomyopathy
HD	Huntington’s disease
HIF-1α	Hypoxia-inducible factor 1α
Hch1	High copy Hsp90 suppressor 1
HIP	Hsp70-interacting proteins
HSPs	Heat shock proteins
Hsp90	Heat shock protein 90
IGF	Insulin-like growth factors
JNK	c-Jun N-terminal kinase
LRP1	Low-density lipoprotein receptor-related protein-1
MD	Middle domain
MDR	Multidrug resistance
NTD	N-terminal nucleotide-binding domain
PD	Parkinson’s disease
PP5	Protein phosphatase 5
PTMs	Post-translational modifications
PPP	Pentose phosphate pathway
PPIase	Peptidyl-prolyl cis-trans isomerase
PIKK	Phosphatidylinositol-3 kinase–related kinase
PR	Progesterone receptor
PTP	Mitochondrial permeability transition pore
ROS	Reactive oxygen species
SREBPs	sterol regulatory element-binding proteins
SDH	Succinate dehydrogenase
SHR	Steroid hormone receptor
TAC	Transverse aortic constriction
TCA	Tricarboxylic acid
TRAP1	Tumor necrosis factor receptor-associated protein
TPR	Tetratricopeptide repeat
TLR	Toll-like receptors
UPR	Unfolded protein response
VEGFRs	Vascular endothelial growth factor receptors
